# Examining the Effectiveness of Interactive Webtoons for Premature Birth Prevention: Protocol for a Randomized Controlled Trial

**DOI:** 10.2196/58326

**Published:** 2024-05-15

**Authors:** Sun-Hee Kim, Jennie C De Gagne

**Affiliations:** 1 College of Nursing Research Institute of Nursing Science Daegu Catholic University Daegu Republic of Korea; 2 School of Nursing Duke University Durham, NC United States

**Keywords:** cartoon, cartoons, webtoon, webtoons, story, stories, storytelling, preterm, infant, infants, infancy, baby, babies, neonate, neonates, neonatal, newborn, newborns, perception, perceptions, web-based, satisfaction, client satisfaction, clinical trial protocol, education, health belief model, web-based intervention, premature birth, prevention and control, prevention, premature, maternal, pregnant, pregnancy, randomized controlled feasibility trials, self-efficacy, women, randomized, controlled trial, controlled trials, birth, mobile phone

## Abstract

**Background:**

Premature birth poses significant health challenges globally, impacting infants, families, and society. Despite recognition of its contributing factors, efforts to reduce its incidence have seen limited success. A notable gap exists in the awareness among women of childbearing age (WCA) regarding both the risks of premature birth and the preventative measures they can take. Research suggests that enhancing health beliefs and self-management efficacy in WCA could foster preventive health behaviors. Interactive webtoons offer an innovative, cost-effective avenue for delivering engaging, accessible health education aimed at preventing premature birth.

**Objective:**

This protocol describes a randomized controlled trial to assess the effectiveness and feasibility of a novel, self-guided, web-based intervention—Pregnancy Story I Didn’t Know in Interactive Webtoon Series (PSIDK-iWebtoons)—designed to enhance self-management efficacy and promote behaviors preventing premature birth in WCA.

**Methods:**

Using an explanatory sequential mixed methods design, this study first conducts a quantitative analysis followed by a qualitative inquiry to evaluate outcomes and feasibility. Participants are randomly assigned to 2 groups: one accessing the PSIDK-iWebtoons and the other receiving Pregnancy Story I Didn’t Know in Text-Based Information (PSIDK-Texts) over 3 weeks. We measure primary efficacy through the self-management self-efficacy scale for premature birth prevention (PBP), alongside secondary outcomes including perceptions of susceptibility, severity, benefits, and barriers based on the health belief model for PBP and PBP intention. Additional participant-reported outcomes are assessed at baseline, the postintervention time point, and the 4-week follow-up. The feasibility of the intervention is assessed after the end of the 3-week intervention period. Outcome analysis uses repeated measures ANOVA for quantitative data, while qualitative data are explored through content analysis of interviews with 30 participants.

**Results:**

The study received funding in June 2021 and institutional review board approval in October 2023. Both the PSIDK-iWebtoons and PSIDK-Texts interventions have been developed and pilot-tested from July to November 2023, with the main phase of quantitative data collection running from November 2023 to March 2024. Qualitative data collection commenced in February 2024 and will conclude in May 2024. Ongoing analyses include process evaluation and data interpretation.

**Conclusions:**

This trial will lay foundational insights into the nexus of interactive web-based interventions and the improvement of knowledge and practices related to PBP among WCA. By demonstrating the efficacy and feasibility of a web-based, interactive educational tool, this study will contribute essential evidence to the discourse on accessible and scientifically robust digital platforms. Positive findings will underscore the importance of such interventions in fostering preventive health behaviors, thereby supporting community-wide efforts to mitigate the risk of premature births through informed self-management practices.

**Trial Registration:**

Korea Disease Control and Prevention Agency (KDCA) KCT0008931; https://cris.nih.go.kr/cris/search/detailSearch.do?seq=25857

**International Registered Report Identifier (IRRID):**

DERR1-10.2196/58326

## Introduction

### Background

Premature birth is defined as a live birth that occurs before 37 weeks of pregnancy. The estimated global premature birth rate per 100 live births was 9.9% (13.4 million premature births) in 2020 [1]. It poses a serious health issue for children, their families, and society worldwide [2], with complications from premature birth being the primary contributors to childhood mortality. This accounts for 35% of all deaths during the neonatal period and 18% of all deaths in children younger than 5 years [3]. Premature birth is also associated with short-term and long-term morbidity, developmental loss in children, adult-onset diseases, and negative impacts on human capital, such as lower education and income levels and reduced social success [2].

A variety of community, societal, maternal, and fetal health factors affecting premature birth before and during pregnancy have been identified [2]. Interventions including medication, surgical procedures, cervical devices, targeted diets, physical exercise, smoking cessation programs, nutritional supplementation, education, and various special tests or investigations have been developed with reported effectiveness [4,5]. However, despite efforts to reduce its prevalence over the past several decades, the rate of premature birth has not significantly changed, showing a slight increase of 0.14% per year on average from 2010 to 2020 [1]. Women of childbearing age (WCA) still lack awareness about premature birth and the necessary health behaviors to prevent it [6,7]. Research has predominantly focused on the effects of trials with single-pronged interventions targeting narrow aspects, unable to address the complexity of premature birth’s multifactorial etiological network of risk factors [2]. Moreover, step-by-step, long-term interventions are recommended for women both before and during pregnancy, with most intervention development concentrating on pregnant women and neglecting WCA in prepregnancy stages [7].

To prevent premature birth, it is crucial for women to be aware of its risks and to cultivate preventive health behaviors. The health belief model highlights the importance of strengthening health beliefs, which include perceived susceptibility, severity, benefits, and barriers, alongside self-efficacy and behavioral intention, in promoting health behaviors [8]. Studies have shown that health beliefs significantly influence prepregnant [9] and pregnant women [10-13], predicting health behavior. Self-efficacy is a key factor in WCA’s health behaviors before [7,9] and during pregnancy [13]. Thus, enhancing health beliefs and self-efficacy regarding premature birth prevention (PBP) could lead to an increase in preventive health behaviors.

WCA have shown a preference for web-based education for its accessibility and convenience [7]. Systematic reviews have indicated the effectiveness of web-based education programs for WCA both before [14] and during pregnancy [15,16], suggesting web-based interventions as practical and potentially more effective educational tools. Such interventions, adaptable across devices, offer personalized and interactive educational experiences [16]. Moreover, WCA have expressed a preference for case-based education using visuals over text [7].

Webtoons, digital comics, or graphic novels presented in a vertical, scrollable format designed for easy reading on computers and mobile devices contain narrative, image, metaphor, and characterization elements. They are an effective educational method that increases understanding and awareness, easily creates empathy, and engages readers through interactive storytelling [17]. Webtoons have been shown to enhance learners’ interest, concentration, critical thinking, participation, and sharing [17,18]. Their effectiveness in educational interventions, particularly in the context of women’s pregnancy health, has been demonstrated through improvements in actual and perceived knowledge, risk perception, affective assessment, self-efficacy, intention to take protective behaviors, subsequent information-seeking behavior [19], environmental health perceptions, and behaviors during pregnancy [20]. Additionally, webtoons have been effective in increasing self-efficacy and knowledge regarding preventive health management of premature labor among WCA [21]. Therefore, to prepare a new intervention plan that is highly accessible, increases interest and concentration, and allows for participation and sharing to prevent premature birth in WCA, we propose to develop a web-based intervention using webtoons and aim to validate its effectiveness.

### Objectives

This study is designed to evaluate the efficacy and feasibility of “Pregnancy Story I Didn’t Know in Interactive Webtoon Series (PSIDK-iWebtoons),” an interactive web program aimed at preventing premature birth. The efficacy of the intervention will be assessed by comparing self-reported self-management self-efficacy for premature birth prevention (SMSE-PBP), health belief model scale for premature birth prevention (HBM-PBP), and premature birth prevention intention (PBPI) among WCA who receive the PSIDK-iWebtoons intervention to those who receive the text-based version, “Pregnancy Story I Didn’t Know in Text-based Information (PSIDK-Texts).” To explore the feasibility of PSIDK-iWebtoons, we will examine participants’ satisfaction using the Client Satisfaction Questionnaire-8 (CSQ-8) and evaluate the quality of the website, comparing responses between the 2 groups. Additionally, insights into the intervention’s effects and feasibility will be further drawn from a qualitative content analysis based on interviews with participants conducted after the second postintervention period (T2) and from usage data of the intervention recorded through web activity tracking.

## Methods

### Study Design

This study uses an explanatory sequential mixed methods design [22]. Initially, a quantitative study (phase 1) will assess the intervention’s effects and feasibility through a 1:1 parallel-group, superiority, double-blind, randomized controlled trial. Subsequently, phase 2 will involve one-to-one and semistructured interviews to provide deeper qualitative insights.

### Setting

The study will be hosted on a dedicated website titled “Pregnancy Story I Didn’t Know” (Korean: 내가 몰랐던 임신 이야기), with the program referred to as PSIDK-iWebtoons. This platform will be accessible exclusively to invited participants from any region of South Korea, ensuring privacy and control over the study’s environment. A few screenshots of the program displayed on mobile devices are included in Multimedia Appendix 1.

### Inclusion and Exclusion Criteria

Participants will be selected based on the criteria presented in Textbox 1. Participants in this study are WCA who intend to give birth on their own in the future, plan to become pregnant, or are currently in the first trimester of pregnancy, regardless of whether they have high-risk factors for premature birth. Interview participants will be drawn from both the experimental and control groups, focusing on those showing significant changes in main effect variable scores. Approximately 30 participants will be selected for interviews to further explore the intervention’s impact and practicality.

**Inclusion and exclusion criteria for the study.**

**Inclusion criteria**

- Women of childbearing age, ranging from 19 to 49 years
- Women who express intentions to conceive in the foreseeable future, are actively planning for pregnancy, or are currently in the first trimester of pregnancy
- Being able to read and write in Korean
- Having access to an intern-capable device (smartphone, tablet, laptop, or desktop)
- Possessing a valid phone number and email for website account creation
- Active user of mobile instant messaging apps or social media, with readiness to use these for study participation
- Willingness to engage in a self-help web program for 3 weeks and provide written consent for participation

**Exclusion criteria**

- Having a previous experience of premature birth
- Having a career in a childbirth facility as a health care provider

### Intervention

#### Overview

Participants in both the experimental and comparative groups will access their designated web programs using personal internet devices, such as smartphones, tablets, laptops, or desktops. Over a 3-week period, they will independently register by clicking on a provided link, at their convenience, without set limits on duration or frequency of participation. The approach encourages self-guided engagement with the web programs, adhering to principles of minimal contact.

Three days prior to the intervention’s start, participants will receive an SMS text message on their mobile phone from a research assistant, outlining details about the commencement and procedures for participation. On the morning of the start date, an additional message will provide specific information, including the website address, the participation time frame, account creation instructions, an introduction to the program, learning methodologies, reflection writing guidelines, and a note to prevent account sharing. Further, reminder messages will be sent twice a week (Monday and Thursday) after the intervention begins. Participants found engaging in activities not related to the intervention’s objectives, such as marketing promotions or violating digital etiquette and civility standards, will be subject to removal by the administrator according to the established discontinuation criteria.

#### Experimental Group

##### Overview

Participants in the experimental group will engage with the PSIDK-iWebtoons intervention through the website. Designed as a responsive web application, it aims to improve accessibility across different operating systems and devices, catering to a wide range of users. The intervention includes 7 webtoon series coupled with professional information, and it encourages participants to make comments or provide feedback by writing about their reflections on the webtoon series.

##### Webtoon Series Learning and Repeated Learning

The webtoon series features the stories of 7 women, 6 of whom have high-risk factors for premature birth, spanning from prepregnancy to pregnancy phases, with each story culminating in 3-4 different endings. Participants in the experimental group begin by creating personal accounts and completing questionnaires to identify high-risk factors for premature birth (Textbox 2). Based on the questionnaire responses, a personalized list of recommended webtoon series is generated and prioritized at the top of the web page for easy selection.

**Questionnaires on high-risk factors for premature birth.**

Please respond with “yes” if applicable, “no” if not applicable, or “not sure” by checking the appropriate box next to each of the following risk factors for premature birth.

- Are you able to manage your pregnancy health effectively on your own?
- Have you undergone prepregnancy health checkups? Or do you intend to do so?
- Is your current age or planned age at the time of pregnancy 36 years or older?
- Were all your pregnancies planned, or do you intend for future pregnancies to be planned?
- Do you currently smoke?
- Do you handle stress well, or do you have difficulty managing stress?
- Do you have many responsibilities, such as work, childcare, and caregiving?
- Have you had difficulty with nutritional intake or been underweight currently or before pregnancy?
- Are you currently overweight or obese currently or before pregnancy?
- Have you experienced premature labor in the past?
- Have you been trying to conceive for over a year without success, experiencing infertility?
- Do you currently have high blood pressure, or did you have hypertension during a past pregnancy?
- Have you been diagnosed with diabetes or gestational diabetes currently or in the past?
- Do you currently have or have you ever had amniotic fluid leak before 37 weeks of pregnancy?
- Have you had cervical surgery currently or in the past?
- Have you experienced early separation of the placenta before childbirth, causing bleeding?
- Do you currently have excess amniotic fluid, or did you have it in the past?
- Are you currently pregnant with twins, or have you had a twin or more pregnancies in the past?
- Are you frequently exposed to chemicals or pesticides?
- Do you have uterine fibroids (myomas)?

Participants are then guided through an introduction to the program’s objectives and how to navigate the website efficiently. This includes accessing the PBP education page to choose and read from the series recommended to them or from the entire list of webtoon series available. Each story is designed with key learning outcomes in mind and incorporates 2 or 3 decision-making user interfaces that allow participants to direct the actions of the protagonist. The choices made at these decision points alter the course of the story, leading to various endings. Each webtoon series is structured to have 3 or 4 possible outcomes, enriching the learning experience by illustrating the consequences of different health decisions and behaviors.

Participants are unrestricted in their choice of webtoon series from the list, with no limits on viewings, fostering repeated engagement. After finishing a story ending, they are taken to the webtoon list on their “my page,” now updated with a badge marking completion and the completion date. The “my page” learning record will then list the series engaged with, showing high-resolution, clear images for endings they have completed. Conversely, endings that remain unexplored are indicated by blurred images, marked “Incomplete.” These blurred images visually cue participants about content that awaits their attention. An indicator of learning progress is also displayed as a percentage for each ending, allowing for a review of choices made and the option to continue from the last decision point, enhancing the interactive and personalized learning experience.

##### Professional Information Review

Participants in the experimental group have access to detailed professional information linked to the learning content of the webtoons. This information is succinctly summarized and available in 2 formats: “Learn All” and “Learn Selectively.” “Learn Selectively” allows participants to delve into health information pertinent to each individual series upon its completion, while “Learn All” offers comprehensive insights across all series, accessible at any point during the intervention. This structured approach enables participants to either broaden their knowledge base or focus on specific topics of interest.

##### Writing and Sharing Reflections

Following the exploration of each webtoon series, participants are encouraged to write reflections. These reflections prompt them to answer semistructured questions on various aspects, including the main narrative, the most impactful content, learned health behaviors for PBP, strategies for coping with PBP, and areas of confusion or curiosity. Reflections are to be concise, with a length requirement of 30-300 Korean characters (roughly equivalent to 20-200 English words), ensuring focused and meaningful contributions. At least one reflection must be shared publicly, fostering an interactive community environment. Participants can then view and engage with others’ reflections, leaving comments and expressing appreciation through “hearts” (emoticons), further enhancing the communal learning experience.

#### Comparative Group

To maintain the integrity of the study through blinding, a specialized website was developed exclusively for the comparative group. This platform, hosted on TISTORY—a blogging service by Kakao Corp—mimics the layout of conventional websites, featuring primarily text-based content. This choice leverages the ubiquity of KakaoTalk (Kakao Corp), a messaging service dominating 98% of messaging app usage time in Korea, to ensure familiarity and accessibility for participants [23].

Participants in this group will be introduced to the PSIDK-Texts intervention. Unlike their counterparts in the experimental group, who interact with the webtoon series, the comparative group will engage with 7 text-based fictional cases. These narratives mirror the webtoon content available to the experimental group, ensuring content parity across groups. Additionally, they will have access to the same professional information, available in both “Learn Selectively” and “Learn All” formats, aligning with the educational material provided to the experimental group.

The engagement process for the comparative group involves creating a personal account on the TISTORY platform. From there, participants will navigate the intervention autonomously, reading through the case studies and accompanying professional information. They are also encouraged to reflect on their learning by writing and sharing at least one reflection on the platform, mimicking the reflective exercise of the experimental group but within a text-centric learning environment.

Upon the conclusion of the final survey, participants in the comparative group will be granted access to the same educational program that was provided to the experimental group. They will receive the URL, enabling them to benefit from the program at their convenience, thereby ensuring equitable access to the intervention resources after trial.

### Outcomes

#### Primary Outcome: SMSE-PBP

The primary quantitative outcome is measured by the SMSE-PBP, encompassing 3 dimensions across 10 subscales with a total of 60 items, validated for reliability [24]. The SMSE-PBP spans prepregnancy (dimension 1) with a 3-factor scale of 13 items, pregnancy (dimension 2) with a 5-factor scale of 32 items, and hospital stay (dimension 3) with a 2-factor scale of 15 items [24]. This scale was selected due to the absence of existing measures for behaviors related to PBP and the critical role of self-efficacy in health behavior [25]. Responses are gauged on a 5-point Likert scale from 1=I can hardly do it to 5=I can do it very well, with the mean of the total item scores ranging from 1 to 5. Scores for prepregnancy, pregnancy, and hospital phases indicate respective levels of self-management self-efficacy, with higher scores reflecting greater efficacy. At the time of development, Cronbach α was reported as 0.88 for dimension 1, 0.96 for dimension 2, and 0.96 for dimension 3 [24].

#### Secondary Outcomes

##### Health Belief Model Scale for Premature Birth Prevention

This scale assesses perceived susceptibility, severity, benefits, and barriers regarding premature birth, featuring 4 dimensions across 15 subscales with 96 items, validated for reliability [26]. The number of items in the 4 dimensions of the HBM-PBP is as follows: perceived susceptibility (21 items), severity (26 items), benefits (27 items), and barriers (22 items).

Each item on this scale is rated using a 5-point Likert scale that ranges from 1=not likely at all to 5=very likely. The scores for each dimension are computed as the mean of the total item scores, ranging from 1 to 5. Higher scores across these dimensions indicate stronger health beliefs and perceptions concerning the susceptibility, severity, benefits, and barriers of premature birth. At the time these measures were developed, the Cronbach α values were established as 0.87 for perceived susceptibility, 0.94 for perceived severity, 0.94 for perceived benefits, and 0.92 for perceived barriers [26].

##### Premature Birth Prevention Intention

The PBPI score assesses participants’ intentions toward preventing premature birth. This instrument was specifically developed for this trial, drawing upon previous research on prevention intention. Participants respond to 3 statements: “I intend to do something to prevent premature birth,” “I plan to do something to prevent premature birth,” and “I will try to do something to prevent premature birth.” Responses are captured on a 7-point Likert scale from 7=strongly agree to 1=strongly disagree. The mean of the total item scores, ranging from 1 to 7, reflects the intensity of the participant’s prevention intention, with higher scores indicating stronger intentions toward preventing premature birth.

#### Feasibility Outcomes

##### Client Satisfaction Questionnaire-8

Participant satisfaction with the web-based education program will be measured using the CSQ-8, developed by Attkisson and Zwick [27], which consists of 8 items rated on a 1- to 4-point Likert scale; higher aggregate scores indicate greater satisfaction. Criterion validity was tested, and Cronbach α was reported as 0.93 [27].

##### Quality of the Website

The quality of the website used for the study will be assessed with a scale developed by Kang et al [28] and modified by Choi [29], featuring 20 items also rated on a 1- to 4-point Likert scale. A higher score on this scale reflects a more positive evaluation of the website’s quality by users. Content validity was evaluated by experts [28,29] and reported a Cronbach α of 0.82 [29].

##### Use of the Web-Based Intervention

In the experimental group, the use of the web-based intervention will be assessed based on the number of logins during the intervention period, the number of completed series to the end, the number of endings collected for each series, the user’s activities (reflections and comments), and messages to be left in the inquiry. The use of the intervention in the comparison group will be assessed based on users’ reflections on the educational program in the guestbook.

#### Qualitative Outcome

This study will gather qualitative insights using a semistructured interview to complement the quantitative data. The primary qualitative outcome will center on an open-ended question that explores participants’ perceived changes in self-efficacy for self-management of PBP before and after engaging with the education program. Secondary outcomes will delve into participants’ perceptions of susceptibility, severity, benefits, and barriers related to PBP, as framed by HBM-PBP, both pre- and postparticipation. Additionally, open-ended questions concerning satisfaction with the education program and the quality of the website will be posed to assess feasibility outcomes (Multimedia Appendix 2). Follow-up questions will be tailored based on participants’ initial responses to these open-ended inquiries.

#### Demographic Characteristics and Pregnancy- and Birth-Related Characteristics

Demographic characteristics encompass age, education level, occupation, marital status, monthly income, and area of residence. Additionally, pregnancy- and birth-related characteristics data comprise childbirth experience, number of children, diagnosed health problems, prenatal education, self-perceived likelihood of premature birth, level of intention to give birth in the future, and high-risk factors for premature birth.

### Participant Timeline

**Figure 1:**
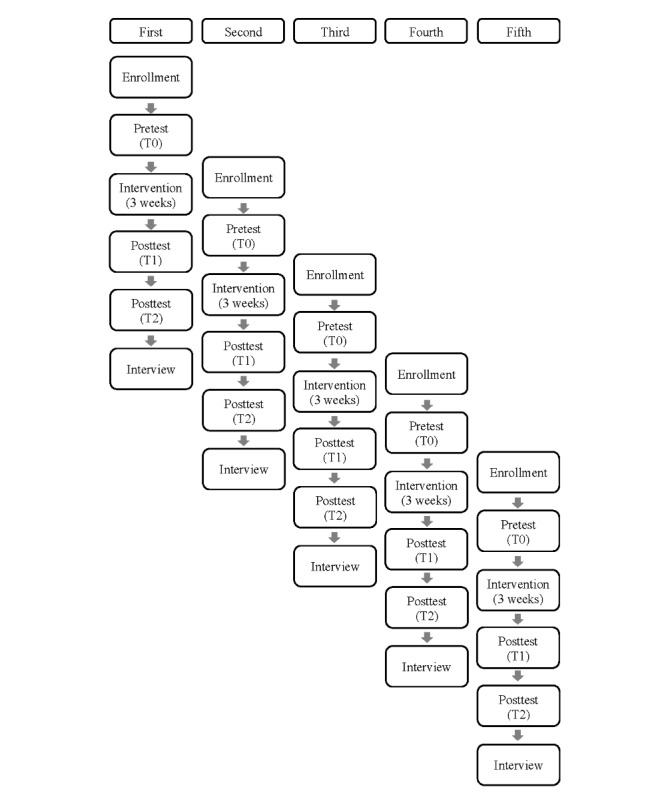
Time schedule of enrollment, interventions, assessments, and interviews for participants.

Recruitment and intervention delivery for participants are structured over 5 cycles. Each recruitment phase spans 2 weeks, during which interested individuals can sign up and immediately complete a preintervention digital survey. After this period, followed by another 2 weeks, an intervention link is sent via SMS text message 3 days prior to commencement (typically on Friday), signaling the start of a 3-week engagement phase according to each participant’s schedule. Throughout the intervention, SMS text messages from research assistant A are sent twice a week (on days 1, 4, 8, 11, 15, and 18) to encourage ongoing participation. The notification schedule is designed to avoid weekends and provide notifications in the middle of the day. A postintervention survey (T1) invitation is sent via text shortly after the intervention ends, with participants expected to complete this survey autonomously within the following week. After a 4-week washout period, a second postintervention survey (T2) follows a similar process. Subsequently, participants are invited to partake in one-on-one qualitative interviews, conducted on the web by 2 researchers. Each recruitment cycle initiates after the completion of the preceding 2-week recruitment phase. A detailed timeline of enrollment, interventions, assessments, and interviews is illustrated in Figure 1.

### Sample Size

The sample size was calculated using G*Power software (Heinrich Heine University) [30], considering the absence of identical measurement tools and programs for an independent *t* test. A medium effect size of 0.50, a 1-sided significance level of 0.025, a power of 0.80, and an equal allocation ratio (1:1) for the 2 groups were assumed. This analysis determined that a total of 128 participants are required. Given the dropout rates of 20%-40% reported in previous web education program research [31,32], we anticipate a 30% dropout rate. To accommodate this, the study aims to enroll a total of 182 participants, allocating 91 participants to both the experimental and comparative groups, respectively.

### Recruitment

The recruitment strategy includes using social media platforms such as Twitter, digital forums related to pregnancy or marriage, Kakao open chat rooms, and lifestyle information apps like Daangn Market Inc. Interested individuals will follow the provided web link to read the study details and consent form. The eligibility screening will include questions about gender, age, childbearing intentions, experience with premature birth, and experience working in obstetric clinical settings. Eligible participants will then complete a preintervention survey.

### Allocation

Upon consenting to participate, individuals will receive an alphanumeric identification code from research assistant B, who will also stratify participants by childbirth experience. Allocation sequences will be generated using the “randombetween (1,6)” formula in Microsoft Excel (Microsoft Corp) with 4-block randomization, assigning participants to 1 of 2 groups. Two days before the intervention starts, research assistant A will inform participants of their group’s specific URL. Participants will remain unaware of their group allocation on the first day of the intervention, with the allocation process being managed by research assistant B.

### Blinding

Participants will be informed at the onset that they will be randomly assigned to 1 of 2 web-based interventions, with the design making it challenging for them to discern their assigned intervention. This ensures participants are blinded to their group allocation throughout the intervention, postintervention surveys, and interviews. The comparative group will only be unblinded after completing the interview phase, at which point they will be offered the intervention provided to the experimental group. Research assistant B will be temporarily hired solely for participant allocation, and the other researchers, including research assistant A, will not participate in the allocation process.

### Data Collection Methods

#### Survey Data

Outcomes and demographic and pregnancy- and birth-related characteristics data collection will be managed on the web by research assistant A, using survey URLs sent to participants. To ensure comprehensive data collection, during the posttest phases T1 and T2, participants will receive reminder SMS text messages on the first, third, and sixth days (for nonrespondents), prompting completion of the survey. Additionally, use data will be systematically gathered from the web program’s administrative interface. These data will be requested at regular intervals throughout the study to ensure comprehensive coverage and accuracy in our analysis of participant engagement.

#### Interview Data

To enrich the quantitative findings and delve into barriers to intervention participation, qualitative interviews will be conducted with 30 participants, selected purposefully to represent diverse experiences across both experimental and comparison groups and varying outcomes. These semistructured interviews will be carried out by the research team via Zoom (Zoom Video Communications, Inc) or KakaoTalk, aiming for an insightful blend of video and voice meetings. Immediate transcription will follow each recorded interview, with data analysis facilitated by NVivo software (version 14 for Windows; Lumivero). Prior to interviews, investigators will familiarize themselves with participants’ demographic, baseline, and both T1 and T2 data. Training for interviewers will emphasize techniques for fostering a comfortable atmosphere, building rapport, maintaining neutrality, and eliciting detailed responses. The research team will hold weekly debriefing sessions to discuss any new findings or issues encountered during data collection.

### Data Management

To safeguard participant confidentiality, unique identification numbers will anonymize all data, which will be encrypted and stored in a nonidentifiable format. These data, alongside automatically generated identification codes, will be kept on a dedicated USB storage device and secured in locked filing cabinets, accessible solely to the designated data analyst. In line with Daegu Catholic University’s research data management policy, all data will be retained by the principal researcher (SHK) for 3 years after study completion. Subsequent to this period, all documents will be destroyed, and digital files permanently erased to ensure irretrievability.

### Data Analysis

#### Quantitative Data Analysis

The quantitative data collected will be processed using SPSS Statistics (version 25.0; IBM Corp). Descriptive statistics, including frequency, percentage, mean, and SD, will evaluate demographic and pregnancy- and birth-related characteristics. The analysis of primary, secondary, and feasibility outcomes will also include mean, SD, and tests for normal distribution. To compare the homogeneity of characteristics and outcomes (T0) between experimental and comparative groups, chi-square tests and 2-tailed *t* tests will be applied. Depending on the homogeneity of baseline characteristics and initial SMSE-PBP scores, either repeated measures ANOVA or repeated measures analysis of covariance will be used. For nonnormally distributed data, generalized estimating equations will be used. Subgroup analyses based on demographic and pregnancy- and birth-related characteristics (eg, age and education level) will be conducted using linear or logistic regression. The use of the web-based intervention (the number of logins during the intervention period, the number of series completed to the end, and the number of endings collected for each series) in the experimental group will be assessed for primary, secondary, and feasibility outcomes using linear regression analysis. The reliability of measurement tools will be assessed using Cronbach α coefficient, and missing data will be addressed through multiple imputation.

#### Qualitative Data Analysis

Interview data will be explored starting with the frameworks of SMSE-PBP, HBM-PBP, CSQ-8, and website quality evaluation. Using the qualitative content analysis method by Hsieh and Shannon [33], the process begins with enhancing sensitivity to the data through repeated listening to recordings and reviewing transcriptions. Follow-up interviews may clarify ambiguities, with field notes enhancing content accuracy. Familiarization with the transcribed content aims to deeply understand participant experiences and gain insights into the intervention’s impact. Initial coding involves identifying keywords and phrases from the text, generating preliminary codes from the first few participants, and iteratively integrating new and overlapping codes as the participant pool expands. Codes will be organized into categories, examining their interrelations and distinctions, with themes emerging to represent recurring concepts or latent ideas. To validate the analysis methods and findings, discussions among researchers, reviews by 2 professors, and feedback from 5 experienced qualitative researchers will ensure the derived data and themes are meaningful and accurately represented. For the validation and reliability of the qualitative components of our study, the research team will develop a reference framework. This framework will be based on the subcategories identified within the SMSE-PBP, HBM-PBP, CSQ-8, and website quality evaluations. To ensure the accuracy of our coding process, data from 1 interview will be coded independently by different researchers. Subsequently, the interpretations of each coding will be discussed collectively among the research team to harmonize understanding and finalize the coding schema. Furthermore, to reinforce the credibility of our findings, transcripts of the interviews will be sent to all participants within 1-3 days following their interview. Participants will be asked to review the content for accuracy and provide any necessary feedback. This member check process is designed to confirm and validate the data captured during the interviews.

### Data Monitoring

The integrity and completeness of data collection will be diligently overseen by research assistant A, with an additional layer of verification provided by the principal researcher. Efforts will be made to address any missing data throughout each survey period, ensuring follow-up does not cause undue distress to participants. As mandated, findings will be systematically reported to the institutional review board of Daegu Catholic University. It is important to note that all aspects of data collection and analysis will be conducted independently of the National Research Foundation of Korea, the sponsoring body. Given the nature of web-based interventions, adverse events are not commonly anticipated. However, should participants experience any unintended effects, they are encouraged to promptly communicate these to the research team for appropriate adjustments. The autonomous participation model of the web-based intervention negates the necessity for regular audit frequencies and procedures by the research team, emphasizing participant-led engagement within the study framework.

### Ethical Considerations

This study protocol has received ethics approval from the institutional review board of Daegu Catholic University (CUIRB-2023-0050). Comprehensive written information about the study will be provided to all participants, from whom informed consent will be obtained prior to their participation. Participation in this study is entirely voluntary and incurs no cost to the participants. All procedures outlined in this protocol will adhere strictly to applicable guidelines and regulations to ensure ethical compliance and participant safety. Addressed within the application were several potential ethical considerations. First, although no adverse effects are anticipated from the web-based intervention itself, it is acknowledged that prolonged screen use may lead to eye fatigue, and participants may incur internet data costs. To mitigate these issues, the research materials will include advice on using a Wi-Fi connection where possible and recommendations against extended continuous screen use. Second, the importance of maintaining participant privacy, especially when using web-based communication for intervention delivery, has been paramount [34]. To safeguard privacy, participants will interact with the study platform using anonymous, auto-generated identification codes. Secure web-based interfaces will be used for all interactions, with only private SMS text messages used for sending reminders to participants. The results of the feasibility study and the broader research findings will be disseminated through various channels to ensure wide reach. These include social networking services, a dedicated project website, and publications in academic journals. This multifaceted approach aims to engage a diverse audience, spanning practitioners, researchers, and the public to maximize the impact of the study’s findings.

## Results

### Development of the Intervention Program

The creation of the PSIDK-iWebtoons website involved a 23-month process that included the writing of scenarios, the production of webtoons, and the development of the website itself. The scenario for PSIDK-iWebtoons was developed by an author who brings a unique blend of expertise: professional experience in nursing care specifically tailored to high-risk pregnant women coupled with a background in scenario writing. This combination ensured the educational content was both medically accurate and engagingly presented. The webtoons were crafted by student interns specializing in webtoon creation, enrolled at Korean universities that offer a major in webtoon studies. The development of the website was managed by a professional company, ensuring both innovative content from emerging artists and a high standard of technical execution. All educational content, including 7 scenarios and webtoon series, underwent 1-2 rounds of expert review by nurses, doctors, and nursing professors. Feedback from WCA was also solicited to refine the scenarios, webtoon series, and website. The intervention scenarios were initially created using insights derived from qualitative interviews with women who have experienced premature births. These initial versions were further refined based on qualitative feedback from 3 WCAs. Additionally, the complete webtoon series and the accompanying website were extensively reviewed by 7 WCAs, who provided both qualitative and quantitative feedback, leading to further revisions.

### Trial Status

The study received funding in June 2021 and institutional review board approval in October 2023. The development of PSIDK-iWebtoons commenced on February 7, 2022, and concluded on November 19, 2023, incorporating enhancements based on pilot testing feedback. The pilot intervention took place from September 4 to 24, 2023, with quantitative data collection occurring from July 17 to October 29, 2023. Qualitative data collection followed from October 30 to November 5, 2023, targeting WCA at high risk for premature birth.

The main quantitative data collection phase started on November 6, 2023, and concluded on March 31, 2024, targeting a broader demographic of WCA. Qualitative data collection for process evaluation commenced in February 2024 and will be completed in May 2024, with analyses and interpretation of these data ongoing. The analysis is expected to be completed by October 2024, and the results will be published by March 2025.

## Discussion

### Expected Findings

This study aims to explore the impact of the PSIDK-iWebtoons intervention, delivered on the web as a self-help tool, on WCA. Anticipated outcomes include improved SMSE-PBP and HBM-PBP, leading to increased engagement in PBP behaviors. Such improvements could contribute to a reduction in premature birth rates. Previous research has highlighted the effectiveness of webtoon educational programs in fostering preventive behaviors to mitigate disease risk [21], suggesting potential benefits for WCA. This research also seeks to evaluate the feasibility of a responsive web program, aiming to expand the knowledge base on interventions effective in preventing premature birth. Given that many WCA are initially unaware of premature birth risks until after giving birth [35], emphasizing preventive measures is crucial. Demonstrating the effectiveness and feasibility of web-based interventions could enhance the accessibility of preventive measures without the constraints of time, location, or direct medical interaction.

### Potential Challenges

Several limitations are anticipated in this study. First, the nature of self-help web-based interventions may result in higher dropout rates. To mitigate this, establishing a notification system that offers personalized content and promotes participant interaction is vital. Due to budget constraints, this study will use manual notifications instead of an automated system. Second, the instruments for measuring primary and secondary outcomes are newly developed. While their psychometric properties have been evaluated, their novelty may influence result validity, necessitating cautious interpretation and the potential use of qualitative data to enrich findings. Third, accurately assessing PBP behaviors presents challenges, and reliance on measures such as SMSE-PBP, HBM-PBP, and PBPI to predict these behaviors has its limitations. Fourth, an unexpected finding that may arise from the intervention is a decrease in the intention to give birth. Research suggests that negative experiences during pregnancy and childbirth can lead some women to reconsider future pregnancies [36]. The webtoon series, designed to realistically portray scenarios involving premature birth, might inadvertently heighten fears associated with such outcomes. This potential influence on WCAs who view the webtoon could lead them to feel apprehensive about the risks of premature birth, impacting their future pregnancy intentions. Fifth, although the comparative group receives a text-based web intervention to compare the effectiveness of story-incorporated cartoons, the scarcity of PBP interventions for WCA may still yield positive outcomes from the text-based approach alone. Additionally, the learning effects across 3 measurements underscore the need for a research design that includes a control group for comparison.

### Conclusions

Preventing premature birth is critical for the health and well-being of children, families, and society at large. Despite the underrecognized importance of PBP, the PSIDK-iWebtoons intervention is designed to raise awareness and underscore the significance of proactive engagement in PBP. By conducting this clinical trial on the web, the study not only assesses the efficacy of the PSIDK-iWebtoons intervention but also highlights its potential to make a positive impact on maternal and child health outcomes.

## Appendix

Multimedia Appendix 1 Screenshots of the webtoon program displayed on mobile devices.

Multimedia Appendix 2 List of the interview questions.

## Abbreviations

CSQ-8: Client Satisfaction Questionnaire-8
HBM-PBP: health belief model scale for premature birth prevention
PBP: premature birth prevention
PBPI: premature birth prevention intention
PSIDK-iWebtoons: Pregnancy Story I Didn’t Know in Interactive Webtoon Series
PSIDK-Texts: Pregnancy Story I Didn’t Know in Text-Based Information
SMSE-PBP: self-management self-efficacy for premature birth prevention
WCA: women of childbearing age

## References

[1] Ohuma EO, Moller AB, Bradley E, Chakwera S, Hussain-Alkhateeb L, Lewin A, Okwaraji YB, Mahanani WR, Johansson EW, Lavin T, Fernandez DE, Domínguez GG, de Costa A, Cresswell JA, Krasevec J, Lawn JE, Blencowe H, Requejo J, Moran AC (2023). National, regional, and global estimates of preterm birth in 2020, with trends from 2010: a systematic analysis. Lancet.

[2] Ashorn P, Ashorn U, Muthiani Y, Aboubaker S, Askari S, Bahl R, Black RE, Dalmiya N, Duggan CP, Hofmeyr GJ, Kennedy SH, Klein N, Lawn JE, Shiffman J, Simon J, Temmerman M (2023). Small vulnerable newborns—big potential for impact. Lancet.

[3] Walani SR (2020). Global burden of preterm birth. Int J Gynaecol Obstet.

[4] Matei A, Saccone G, Vogel JP, Armson AB (2019). Primary and secondary prevention of preterm birth: a review of systematic reviews and ongoing randomized controlled trials. Eur J Obstet Gynecol Reprod Biol.

[5] Medley N, Vogel JP, Care A, Alfirevic Z (2018). Interventions during pregnancy to prevent preterm birth: an overview of Cochrane systematic reviews. Cochrane Database Syst Rev.

[6] Miele MJO, Pacagnella RC, Osis MJD, Angelini CR, Souza JL, Cecatti JG (2018). "Babies born early?"—Silences about prematurity and their consequences. Reprod Health.

[7] Kim SH, Hong JY, Park MK (2022). Educational status and needs of premature birth prevention and its association with preconception health behavior among women of childbearing age in Korea. Res Community Public Health Nurs.

[8] Skinner CS, Tiro J, Champion VL (2015). Health Behavior: Theory, Research, and Practice. 5th Edition.

[9] Malverdy Z, Kazemi A (2016). Health beliefs and stages of changes to improve behaviors among obese and overweight women undergoing preconception care. Iran J Nurs Midwifery Res.

[10] Zambri F, Perilli I, Quattrini A, Marchetti F, Colaceci S, Giusti A (2021). Health Belief Model efficacy in explaining and predicting intention or uptake pertussis vaccination during pregnancy. Ann Ist Super Sanita.

[11] Zambri F, Quattrini A, Perilli I, Spila Alegiani S, Marchetti F, Colaceci S, Giusti A (2022). Health Belief Model efficacy in explaining and predicting intention or uptake influenza vaccination during pregnancy. Ann Ist Super Sanita.

[12] Wahyu D, Kusumaningtyas K, Pratami E (2022). Health education-based effectiveness of Health Belief Model on vulva hygiene behavior in prevention of vaginal discharge for pregnant woman. Open Access Maced J Med Sci.

[13] Mohebbi B, Tol A, Sadeghi R, Mohtarami SF, Shamshiri A (2019). Self-management intervention program based on the Health Belief Model (HBM) among women with gestational diabetes mellitus: a quazi-experimental study. Arch Iran Med.

[14] O'Connor H, Willcox JC, de Jersey S, Wright C, Wilkinson SA (2023). Digital preconception interventions targeting weight, diet and physical activity: a systematic review. Nutr Diet.

[15] Chae J, Kim HK (2021). Internet-based prenatal interventions for maternal health among pregnant women: a systematic review and meta-analysis. Child Youth Serv Rev.

[16] Guo P, Chen D, Xu P, Wang X, Zhang W, Mao M, Zheng Q, Jin Y, Feng S (2023). Web-based interventions for pregnant women with gestational diabetes mellitus: systematic review and meta-analysis. J Med Internet Res.

[17] McNicol S (2017). The potential of educational comics as a health information medium. Health Info Libr J.

[18] Seol YK (2018). Exploration on the applicability of ‘webtoons’ for education: focusing on the aspects of formativity, narrativity and interactivity. Korean J Cult Arts Educ Stud.

[19] Adebayo AL, Davidson Mhonde R, DeNicola N, Maibach E (2020). The effectiveness of narrative versus didactic information formats on pregnant women's knowledge, risk perception, self-efficacy, and information seeking related to climate change health risks. Int J Environ Res Public Health.

[20] Kim HK, Kim HK, Kim M, Park S (2021). Development and evaluation of prenatal education for environmental health behavior using cartoon comics. J Korean Acad Nurs.

[21] Kim SH (2022). Development and effects of a webtoon education program on preventive self-management related to premature labor for women of childbearing age: a randomized controlled trial. Korean J Women Health Nurs.

[22] Creswell JW, Clark VLP (2017). Designing and Conducting Mixed Methods Research. 3rd Edition.

[23] Hong SH (2022). Kakao business expansion through Katalk whose market share is 98% led to the negative effects of monopoly. Dong-A Ilbo.

[24] Kim SH, Lee YJ (2024). Development and validation of a self-management self-efficacy scale for premature birth prevention (SMSE-PBP) for women of childbearing age. BMC Womens Health.

[25] Bandura A (1997). Self-Efficacy: The Exercise of Control. 1st Edition.

[26] Kim SH, Jung SY, Kim Y, Lee YJ (2023). Development and psychometric properties of the health belief model scale for premature birth prevention (HBM-PBP) for women of childbearing age.

[27] Attkisson CC, Zwick R (1982). The client satisfaction questionnaire. Psychometric properties and correlations with service utilization and psychotherapy outcome. Eval Program Plann.

[28] Kang SW, Yoo JS, Ko IS (2005). Development of a health information web site evaluation categories with items for diabetes mellitus. Healthc Inform Res.

[29] Choi YS (2012). The development of web-based ventilator management education program. Journal of the Korea Academia-Industrial cooperation Society.

[30] Faul F, Erdfelder E, Lang AG, Buchner A (2007). G*Power 3: a flexible statistical power analysis program for the social, behavioral, and biomedical sciences. Behav Res Methods.

[31] Batra P, Mangione CM, Cheng E, Steers WN, Nguyen TA, Bell D, Kuo AA, Gregory KD (2018). A cluster randomized controlled trial of the MyFamilyPlan online preconception health education tool. Am J Health Promot.

[32] Gardiner P, Bickmore T, Yinusa-Nyahkoon L, Reichert M, Julce C, Sidduri N, Martin-Howard J, Woodhams E, Aryan J, Zhang Z, Fernandez J, Loafman M, Srinivasan J, Cabral H, Jack BW (2020). Using health information technology to engage African American women on nutrition and supplement use during the preconception period. Front Endocrinol (Lausanne).

[33] Hsieh HF, Shannon SE (2005). Three approaches to qualitative content analysis. Qual Health Res.

[34] Proudfoot J, Klein B, Barak A, Carlbring P, Cuijpers P, Lange A, Ritterband L, Andersson G (2011). Establishing guidelines for executing and reporting internet intervention research. Cogn Behav Ther.

[35] Silva TV, Bento SF, Katz L, Pacagnella RC (2021). "Preterm birth risk, me?" Women risk perception about premature delivery—a qualitative analysis. BMC Pregnancy Childbirth.

[36] Gregory EF, Johnson GT, Barreto A, Zakama AK, Maddox AI, Levine LD, Lorch SA, Fiks AG, Cronholm PF (2024). Communication and birth experiences among Black birthing people who experienced preterm birth. Ann Fam Med.

