# Supplementary material for: Examining the Effectiveness of Interactive Webtoons for Premature Birth Prevention: Protocol for a Randomized Controlled Trial
Source: JMIR Res Protoc. 2024 May 15;13:e58326. doi: 10.2196/58326 (PMC11137428; doi:10.2196/58326)
Supplement: Multimedia Appendix 1 [file resprot_v13i1e58326_app1.pdf]

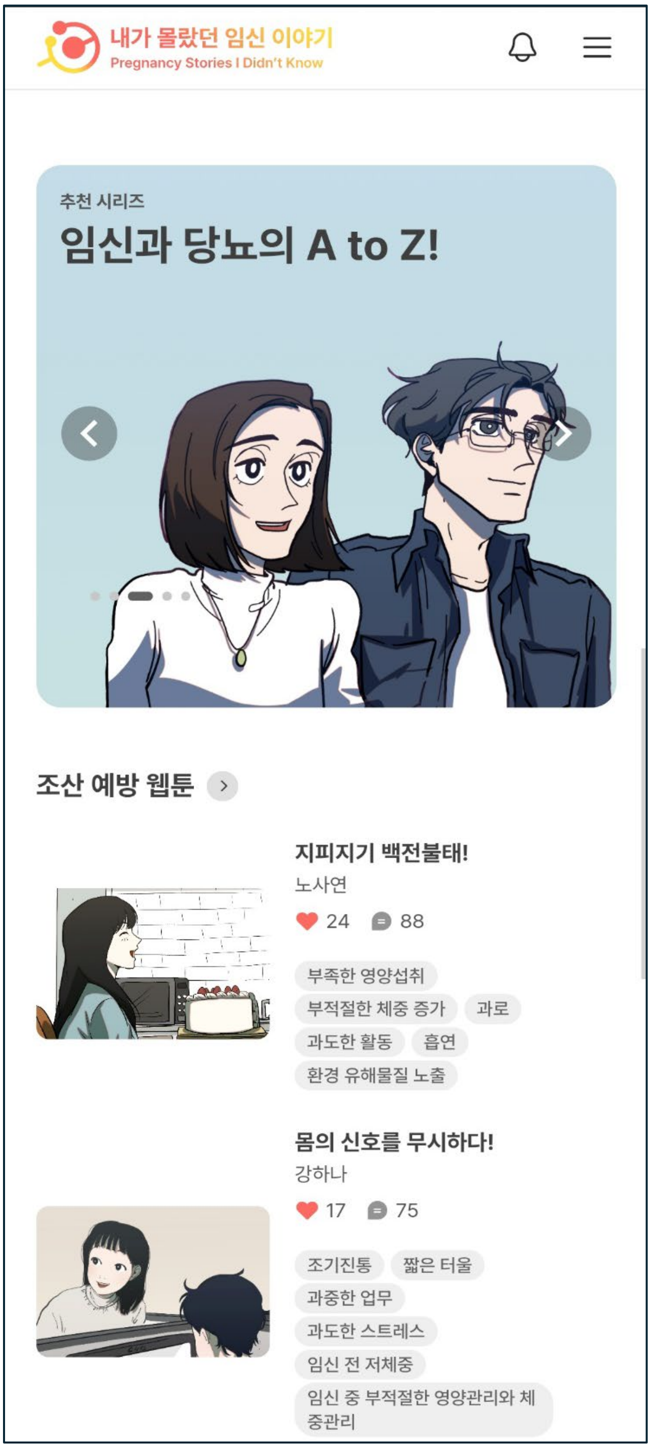

A. Recommend webtoon series.

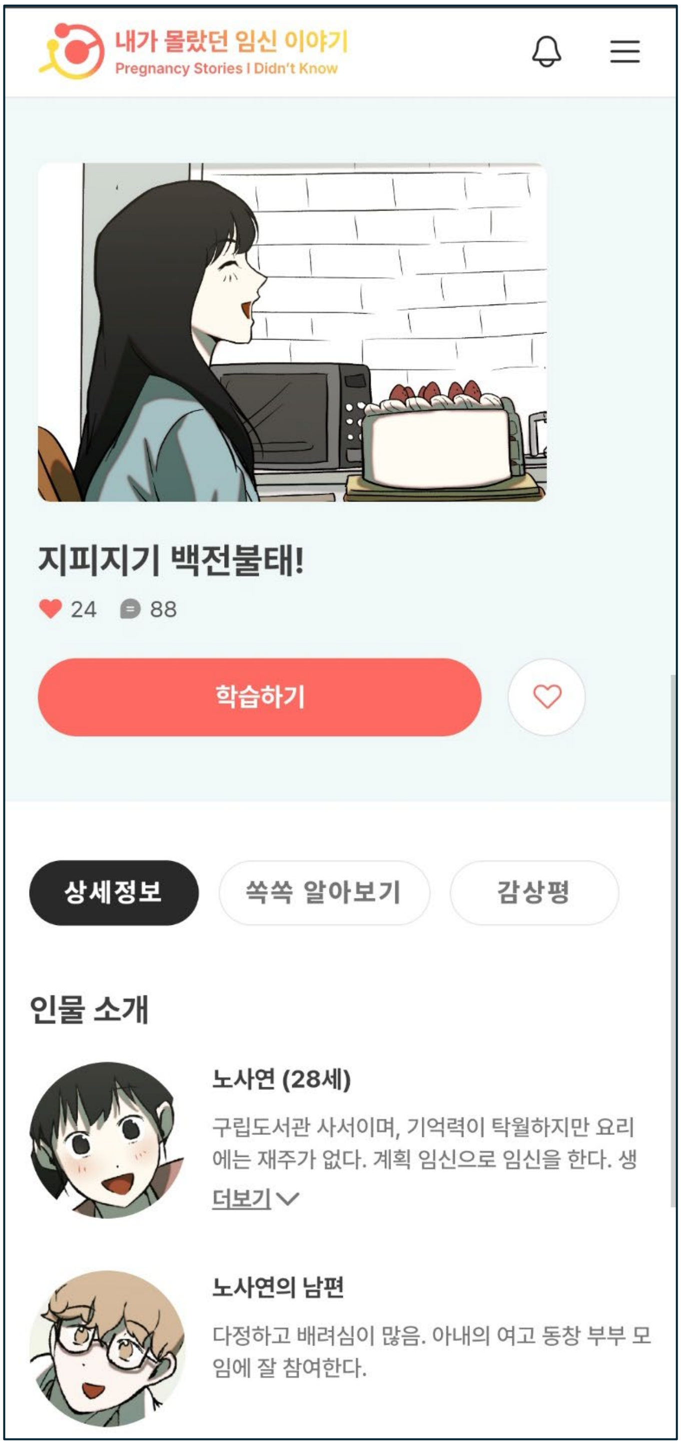

B. An example of a webtoon series.

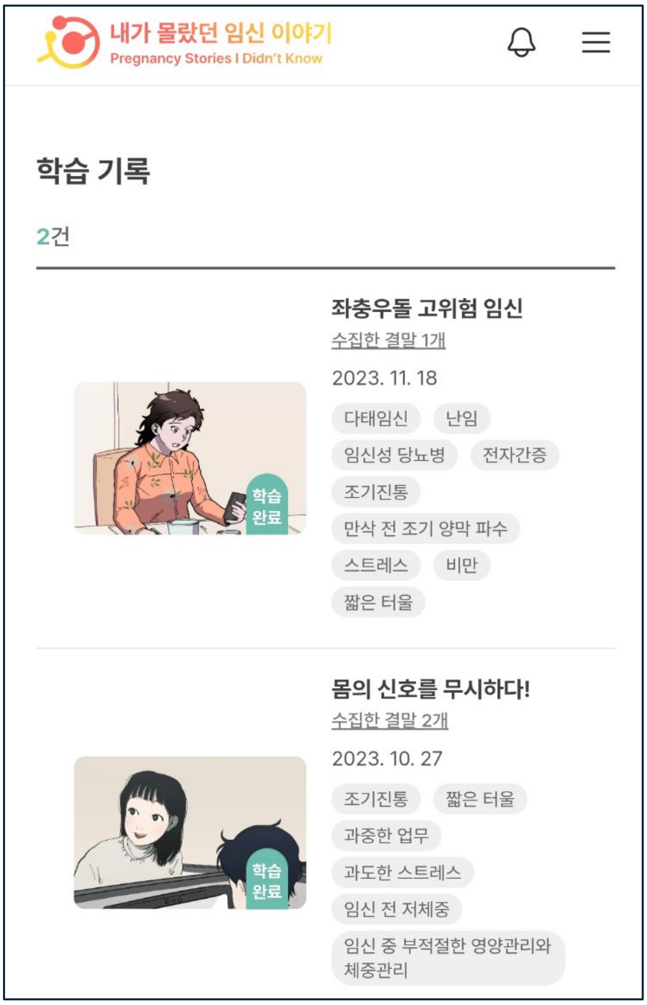

C. User’s learning logs.

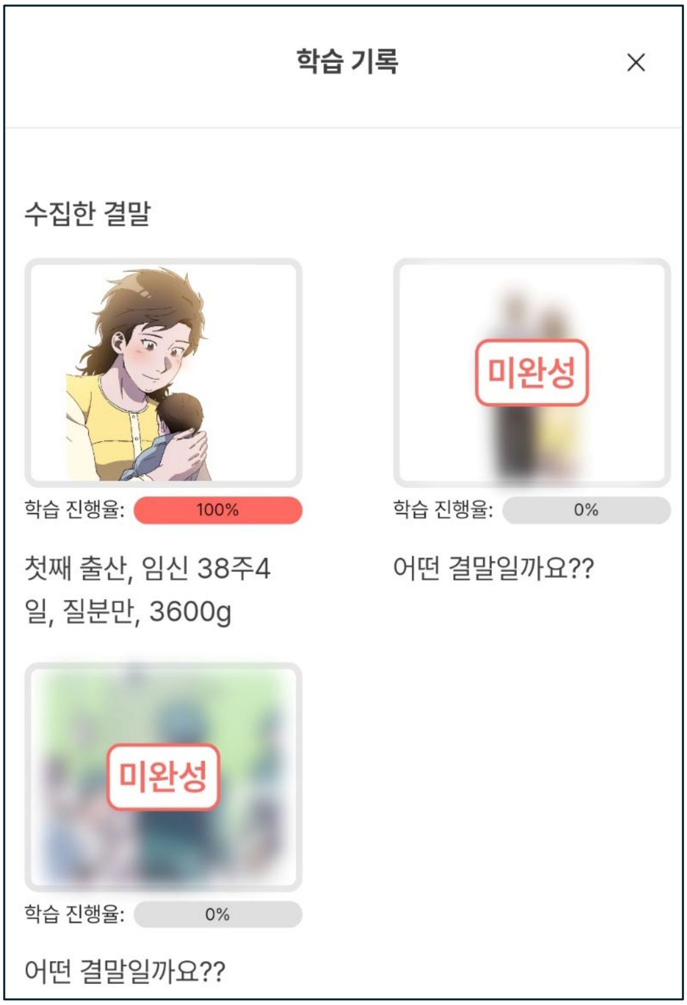

D. Compilation of the episodes of a webtoon series noted in the user’s learning logs
