# Supplementary material for: Examining the Effectiveness of Interactive Webtoons for Premature Birth Prevention: Protocol for a Randomized Controlled Trial
Source: JMIR Res Protoc. 2024 May 15;13:e58326. doi: 10.2196/58326 (PMC11137428; doi:10.2196/58326)
Supplement: Multimedia Appendix 2 [file resprot_v13i1e58326_app2.docx]

| Outcomes | Questions |
| --- | --- |
| SMSE-PBP | How have your perceptions changed before and after participating in the intervention regarding the following:  - I can engage in preventive daily life management before pregnancy.  - I can effectively manage issues preventively before pregnancy.  - I can collaborate well preventively before pregnancy.  - I can manage daily life preventively during pregnancy.  - I can effectively self-monitor for symptoms of risk during pregnancy.  - I can recognize symptoms of risk well during pregnancy.  - I can respond to and manage symptoms well if they occur during pregnancy.  - I can collaborate well preventively during pregnancy.  - I can effectively manage symptoms preventively while hospitalized.  - I can collaborate well with healthcare professionals while hospitalized.  - I can provide preventive support effectively while hospitalized.  - If discharged after being hospitalized during pregnancy, I can manage disease responsively. |
| Perceived susceptibility of HBM-PBP | What is your perception of the likelihood of premature birth? What do you believe are the reasons for this perception? How has your consideration of the possibility of premature birth changed before and after participating in the intervention, based on the following factors:  - Due to inadequate antenatal care  - Due to (in)appropriate handling of premature birth risk  - Depending on the health issues of the pregnant woman  - Based on (in)adequate health lifestyle management of the pregnant woman  - Depending on the psychological health issues of the pregnant woman |
| Perceived severity of HBM-PBP | If you were to experience premature birth, what impacts do you believe might occur?  - Likelihood of worsening emotional well-being due to premature birth  - Possibility of health and developmental issues for the baby resulting from premature birth  - Likelihood of difficulties in daily activities due to premature birth  - Potential for physical health problems resulting from premature birth  - Possibility of physical health problems for the baby resulting from premature birth |
| Perceived benefits of HBM-PBP | What advantages do you believe the following preventive behaviors (preventing premature birth) bring to you? How have these beliefs changed before and after participating in the intervention?  - Health lifestyle management during pregnancy  - Appropriate handling of premature birth risk  - Clinical (medical) management during pregnancy  - Management of risk-promoting factors for pregnancy  - Planned pregnancy |
| Perceived barriers of HBM-PBP | What factors do you believe hinder the following preventive behaviors for premature birth in your case? And why do you think so?  - Appropriate handling of premature birth risk  - Health lifestyle management during pregnancy  - Clinical (medical) management during pregnancy  - Self-management for premature birth risk  - Frequent clinical (medical) management for pregnancy |
| Education program satisfaction | What was the content of PSIDK-iWebtoons (or PSIDK-Texts) intervention like?  Did you get what you wanted from participating in the intervention? What do you think has improved?  What would you like to see improved or added to the intervention?  How much information do you think you gained through the recommended series? The reason is?  How did the recommended webtoon series help you improve your premature birth prevention practices? |
| Website quality evaluation | How was the guidance for website participation?  If there are areas for improvement in overall operation, what would they be?  Having used the website, how would you describe its accessibility and user-friendliness in terms of both access and navigation?  How convenient was the usability of the website?  Did you encounter any errors while using the website?  How would you rate the website's design and layout?  Regarding user feedback collection and overall user experience, including privacy protection, how was the website's performance? What areas do you think could be further improved? |

SMSE-PBP: Self-Management Self-Efficacy Scale for Premature Birth Prevention.

HBM-PBP: Health Belief Model Scale for Premature Birth Prevention.

PSIDK-iWebtoons: Pregnancy Story I Didn't Know in Interactive Webtoon Series

PSIDK-Texts: Pregnancy Story I Didn't Know in Texts-based Information
